# Supplementary material for: Systematic Review and Meta-Analysis on MS-Based Proteomics Applied to Human Peripheral Fluids to Assess Potential Biomarkers of Bipolar Disorder
Source: Int J Mol Sci. 2022 May 13;23(10):5460. doi: 10.3390/ijms23105460 (PMC9141521; doi:10.3390/ijms23105460)
Supplement: Supplementary file 1 [file ijms-23-05460-s001.zip › Supp Info_BD systematic review_meta_analysis_IJMS_2022.pdf]

SUPPLEMENTARY INFORMATION

Systematic Review and Meta-Analysis on MS-Based Proteomics Applied to Human Peripheral Fluids to Assess Potential Biomarkers of Bipolar Disorder

Joao E. Rodrigues [1,2,†], Ana Martinho [1,2,†], Vítor Santos [2,3,4], Catia Santa [1,2], Nuno Madeira [3,4,5], Maria J. Martins [1,2,6], Carlos N. Pato [7], Antonio Macedo [3,4,5,*,‡] and Bruno Manadas [1,2,8,*,‡]

[1] CNC—Center for Neuroscience and Cell Biology, University of Coimbra, 3004-504 Coimbra, Portugal; joao.e.a.rodrigues@gmail.com (J.E.R.); anajmartinho@gmail.com (A.M.); catiajmsanta@gmail.com (C.S.); martins.mjrv@gmail.com (M.J.M.)
[2] CIBB—Centre for Innovative Biomedicine and Biotechnology, University of Coimbra, 3004-504 Coimbra, Portugal; vitorsantos74@gmail.com
[3] Faculty of Medicine of the University of Coimbra, University of Coimbra, 3004-504 Coimbra, Portugal; nunogmadeira@gmail.com
[4] Psychiatry Department, Centro Hospitalar e Universitário de Coimbra, 3004-561 Coimbra, Portugal
[5] CIBIT—Coimbra Institute for Biomedical Imaging and Translational Research, University of Coimbra, 3000-548 Coimbra, Portugal
[6] Medical Services, University of Coimbra Medical Services, 3004-517 Coimbra, Portugal
[7] Department of Psychiatry and Behavioral Sciences, SUNY Downstate Health Sciences University, Brooklyn, NY 11203, USA; carlos.pato@downstate.edu
[8] III Institute for Interdisciplinary Research, University of Coimbra (IIIUC), 3030-789 Coimbra, Portugal

Supplementary Tables

**Table S1**	QUADOMICS criteria to evaluate the quality of the -omics research reports included in a systematic review
**Table S2**	Proteins identified in the selected studies as altered in BD vs. control. Proteins are described by name, UniProt entry name and accession number, and the type of sample (serum, plasma, PBMCs, and saliva). (1) Protein without any information about accession number through UniProt database. Table presented in a supplementary excel file.
**Table S3**	Proteins identified in the selected studies as altered in BD vs. SCZ. Proteins are described by name, UniProt entry name and accession number, and the type of sample (serum, plasma, PBMCs, and saliva). (1) Protein without any information about accession number through UniProt database. Table presented in a supplementary excel file.
**Table S4**	Proteins identified in the selected studies as altered in BD vs. OD. Proteins are described by name, UniProt entry name and accession number, and the type of sample (serum, plasma, PBMCs, whole saliva and sweat). Table presented in a supplementary excel file.
**Table S5**	Results summary from the functional enrichment analysis performed in MetaboAnalyst 5.0. Description of the pathways to which the altered proteins belong to, with indication of number of hits per pathway, as well as statistical analysis of the pathway enrichment and the pathway impact. Table presented in a supplementary excel file.

TABLE S1. QUADOMICS criteria to evaluate the quality of the -omics research reports included in a systematic review

**Study Phase : Phase 1, 2, 3, 4**
1. Were selection criteria clearly described?
2. Was the spectrum of patients' representative of patients who will receive the test in practice? *
3. Was the type of sample fully described?
4. Were the procedures and timing of biological sample collection with respect to clinical factors described with enough detail?<br>4.1. Clinical and physiological factors<br>4.2. Diagnostic and treatment procedures.
5. Were handling and pre-analytical procedures reported in sufficient detail and similar for the whole sample? And, if differences in procedures were reported, was their effect on the results assessed?
6. Is the time period between the reference standard and the index test short enough to reasonably guarantee that the target condition did not change between the two tests?
7. Is the reference standard likely to correctly classify the target condition?
8. Did the whole sample or a random selection of the sample receive verification using a reference standard of diagnosis?
9. Did patients receive the same reference standard regardless of the result of the index test?
10. Was the execution of the index test described in sufficient detail to permit replication of the test?
11. Was the execution of the reference standard described in sufficient detail to permit its replication?
12. Were the index test results interpreted without knowledge of the results of the reference standard?
13. Were the reference standard results interpreted without knowledge of the results of the index test?
14. Were the same clinical data available when test results were interpreted as would be available when the test is used in practice? *
15. Were uninterpretable/intermediate test results reported?
16. Is it likely that the presence of overfitting was avoided?

*Applicable only to phase 3 or 4 studies

Supplementary Figures

**Figure S1**	QUADOMICS evaluation of the quality of the proteomics studies included in the systematic review. QUADOMICS criteria defined in **Supplementary Table S1**.
**Figure S2**	**A)** Publication frequency. Bars show the number of articles published per year using MS-based methods to study BD proteomics. Color reflects the type of sample used: serum, plasma, PBMCs, and saliva. **B)** Number of BD patients in the cohort. The number of bars corresponds to the number of studies published each year, and their height reflects the number of BD patients in the cohort in the study. The average number of BD patients in the cohorts per year is shown in the markers connected by the dashed line.
**Figure S3**	KEGG Mapper Color tool of Focal adhesion. The proteins found in any of the studies are shown in orange, and proteins found to be altered in at least two studies are highlighted in green when the results from the two or more studies are contradictory.

Figure S1

Study	Phase	1	3	4	5	6	7	8	9	10	11	12	13	15	16
Smirnova et al., 2019	2	Yes	Yes	No	Yes	Uncertain	Uncertain	Yes	Uncertain	Yes	No	Uncertain	Yes	Yes	No
Pessoa et al., 2019	2	Yes	Yes	No	Yes	Uncertain	Uncertain	Yes	Uncertain	Yes	No	Uncertain	Yes	Yes	No
Cheng et al., 2018	1	Yes	Yes	No	Yes	Uncertain	Uncertain	Yes	Uncertain	Yes	No	Yes	Yes	Yes	Yes
Petrov et al., 2018	2	Yes	Yes	Yes	Yes	Uncertain	Yes	Yes	Yes	Yes	Yes	Yes	Yes	Yes	No
Knochel et al., 2017	2	Yes	Yes	Yes	Yes	Uncertain	Yes	Yes	Yes	Yes	Yes	Uncertain	Yes	Yes	No
De Jesus et al., 2017	2	No	Yes	No	Yes	Uncertain	Uncertain	No	Uncertain	Yes	No	Uncertain	Yes	Yes	No
Ren et al., 2017	2	Yes	Yes	No	Yes	Uncertain	Yes	Yes	Yes	Yes	Yes	Uncertain	Yes	Yes	No
Song et al., 2015	1	Yes	Yes	Yes	Yes	Uncertain	Yes	Yes	Yes	Yes	Yes	Uncertain	Yes	Yes	No
Chen et al., 2015	2	No	No	No	No	Uncertain	Uncertain	No	Uncertain	No	No	Uncertain	Uncertain	No	No
Giusti et al., 2014	2	Yes	Yes	No	Yes	Yes	Yes	Yes	Yes	Yes	Yes	Uncertain	Yes	Yes	No
Iavarone et al., 2014	2	No	Yes	No	Yes	Uncertain	Uncertain	No	Uncertain	Yes	No	Uncertain	Yes	Yes	No
Herberth et al., 2011	1	Yes	Yes	Yes	Yes	Yes	Yes	Yes	Yes	Yes	Yes	Uncertain	Yes	Yes	Yes
Sussulini et al., 2011	1	No	Yes	Yes	Yes	Uncertain	Uncertain	No	Uncertain	Yes	No	Uncertain	Yes	Yes	No
Sussulini et al., 2010	1	No	Yes	Yes	Yes	Uncertain	Uncertain	No	Uncertain	Yes	No	Uncertain	Yes	Yes	No

Yes
No
Uncertain

Figure S2

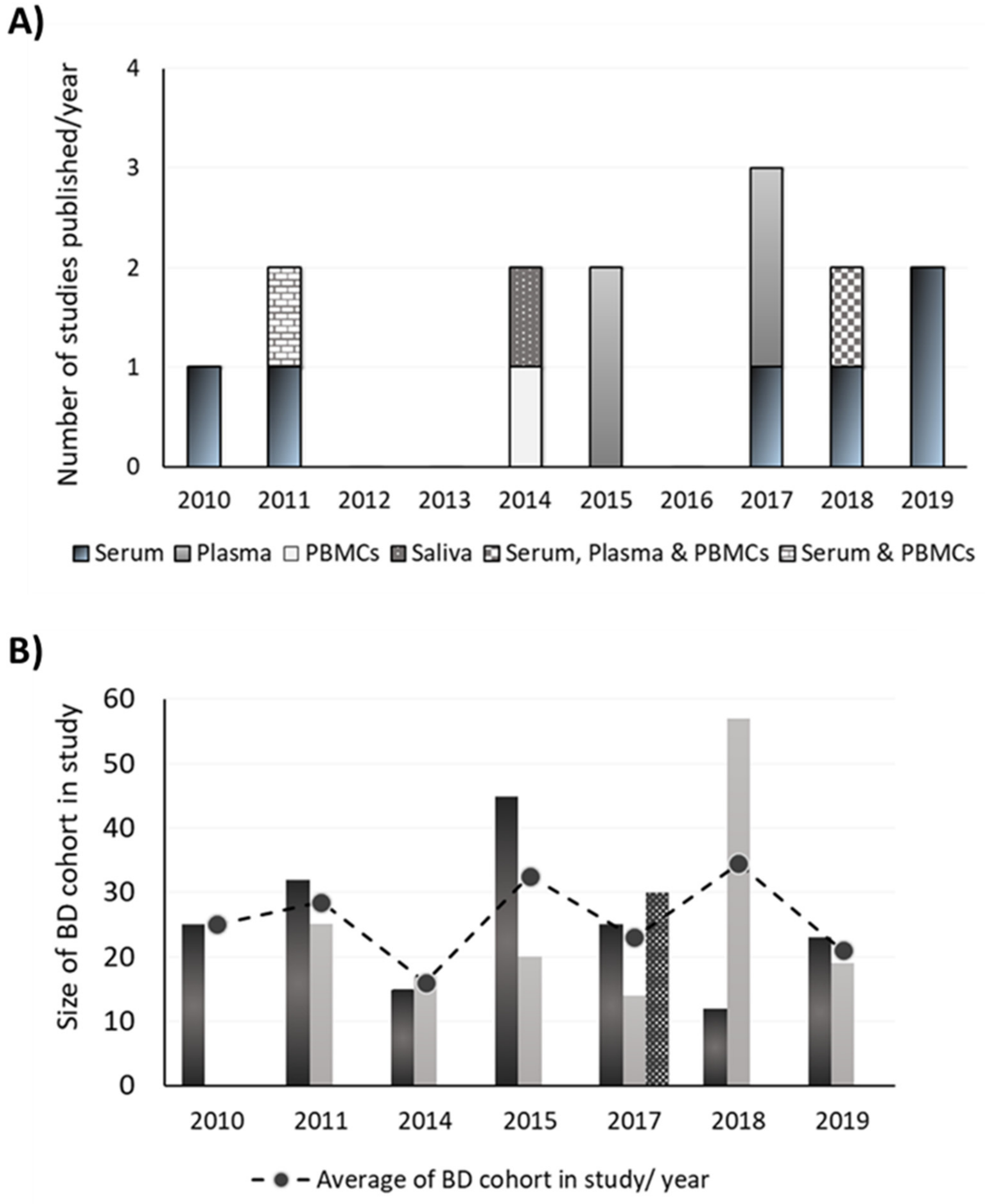
A)
Number of studies published/year
0
1
2
3
4
2010
2011
2012
2013
2014
2015
2016
2017
2018
2019
Serum
Plasma
PBMCs
Saliva
Serum, Plasma & PBMCs
Serum & PBMCs
B)
Size of BD cohort in study
0
10
20
30
40
50
60
2010
2011
2014
2015
2017
2018
2019
Average of BD cohort in study/ year

Figure S3

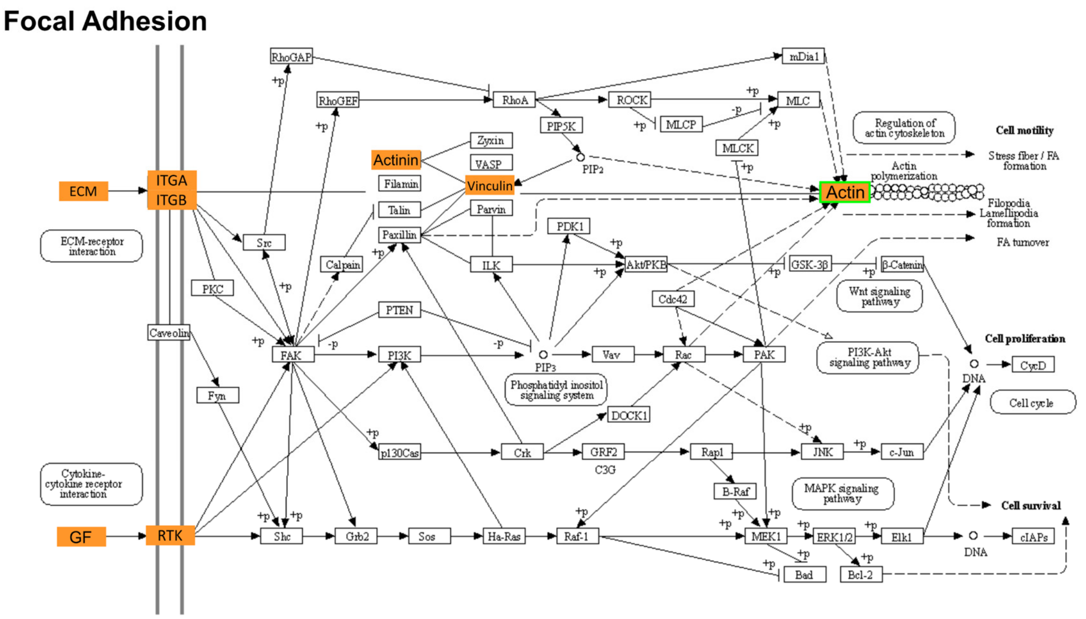

Altered protein in at least one study
Altered protein in at least two studies, with contradictory results
